# Individualized multimodal treatment strategy for metastatic rectal adenocarcinoma: Case report

**DOI:** 10.1016/j.pbj.0000000000000020

**Published:** 2018-07-03

**Authors:** Helena Magalhães, Mário Fontes-Sousa, Manuel Fernandes, Manuela Machado

**Affiliations:** a Medical Oncology Department IPO Porto; b Surgical Oncology Department IPO Porto, Porto, Portugal.

**Keywords:** cytoreduction surgery, multimodal treatment, peritoneal carcinomatosis, rectal cancer

## Abstract

Metastatic rectal cancer requires a multidisciplinary and individualized approach.

The authors describe a case report of a 48-year-old man with recurrence of rectal adenocarcinoma that underwent multimodal treatment, which included chemotherapy with biologic agents, cytoreduction surgery with hyperthermic intraperitoneal chemotherapy, and radiotherapy with improvement in progression-free survival and overall survival.

## Introduction

Peritoneal carcinomatosis from colorectal cancer (CRC) represents a group of patients with metastatic disease associated with poor prognosis.

In metastatic setting, chemotherapy (CTX) eventually with association of biologic agents is the standard of care, with improvement in progression-free survival and overall survival (OS). More recently, there is evidence that cytoreductive surgery combined with hyperthermic intraperitoneal CTX can prolong patients’ life with CRC and peritoneal carcinomatosis.^1^

## Case report

An otherwise healthy, 43-year-old male, with an Eastern Cooperative Oncology Group performance status (ECOG PS) of 0, was admitted in our institution in September 2012 with a recurrence of rectal adenocarcinoma.

Out of our institution, in October 2011, he underwent laparoscopic anterior resection for rectal adenocarcinoma; pathologic stage was pT3N0M0 (6 nodes isolated without disease); no neo or adjuvant therapy was made.

One year later, the patient underwent urgent exploratory laparotomy for intestinal occlusion; a colostomy was made and the diagnosis of peritoneal metastasis was confirmed. Mutational status of the RAS complex was wildtype.

**Figure 1:**
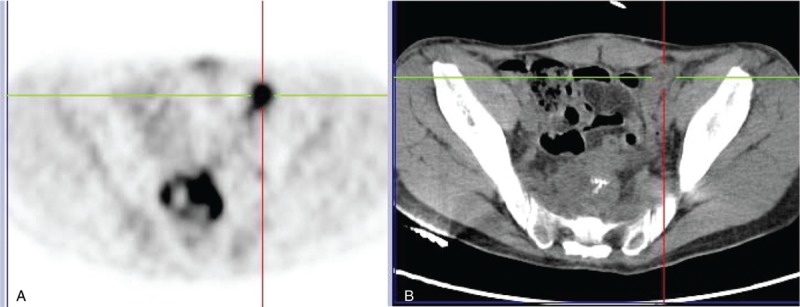
Pelvic tumor implant. (A) FDG-positron emission tomography alone shows a focal area of intense FDG uptake in the left pelvic area, suggesting tumor implant. (B) Computed tomography scan shows the same lesion. FDG = fluorodeoxyglucose.

In our institution, after re-staging with computed tomography (CT) and positron emission tomography–computed tomography (PET–CT), both suggesting local recurrence and pelvic tumor implants (Fig. 1), the case was discussed with multidisciplinary team (MDT), and patient was proposed to CTX with future re-evaluation for cytoreduction surgery with hyperthermic intraperitoneal chemotherapy (CS/HIPEC).

He received 12 cycles of 5-fluorouracil (5-FU), plus irinotecan, plus leucovorin (FOLFIRI) scheme with bevacizumab, without relevant toxicity and with partial response on CT scan and PET–CT (no peritoneal lesions, with persistent presacral tumor but with imagiologic improvement).

In June 2013, CS/HIPEC (mitomycin 80 mg) was performed. The peritoneal cancer index was 11, with apparent complete cytoreduction. There were no postoperative complications. PET–CT was performed with no evidence of disease, and therefore, no complementary CTX was done.

**Figure 2:**
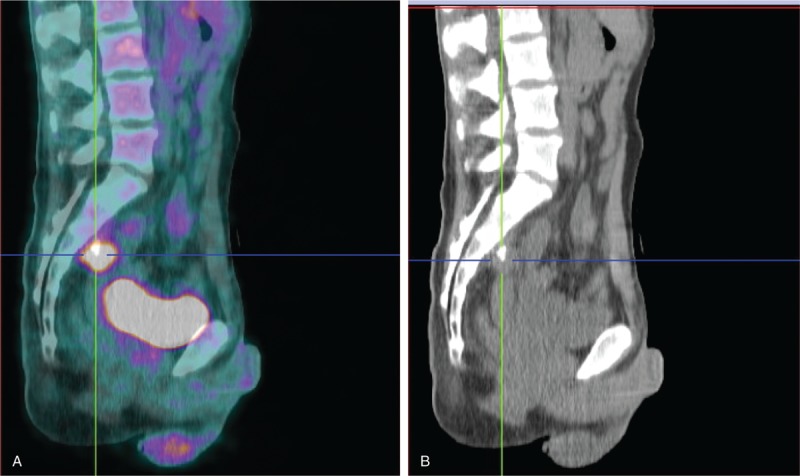
Sagittal images: fused FDG-positron emission tomography/CT (A), CT alone (B). There is increased uptake at the presacral level, suggesting local recurrence, with an SUV_max_ of 6.5. CT = computed tomography, FDG = fluorodeoxyglucose.

Six months later, patient was admitted to the hospital with lumbar pain. An ultrasound and a CT scan only presented right hydronephrosis with no apparent disease recurrence; a percutaneous nephrostomy was made. PET–CT was carried out and revealed increased uptake on the presacral level, suggesting local recurrence (Fig. 2).

After discussion on an MDT, it was decided preoperative concomitant chemoradiation therapy (CRT) with subsequent exploratory laparotomy associated with intraoperative radiotherapy (RT). Patient received 5-FU as radiosensitizer till March 2014.

Eight weeks later, on the exploratory laparotomy, it was identified presacral recurrence and peritoneal carcinomatosis; it was made the resection of the presacral lesion and the reimplantation of the ureter into the bladder, but no intraoperative RT was performed, due to peritoneal disease. The histopathological report confirmed the pelvic recurrence, with positive margins (R1), and the presence of adenocarcinoma in a parietocolic recess.

Patient restarted CTX with the same scheme, 12 cycles, until March 2015, with complete response on CT and PET–CT. MTD suggested a second-look laparotomy, but since he was completely asymptomatic without evidence of disease, it was decided to keep close vigilance.

**Figure 3:**
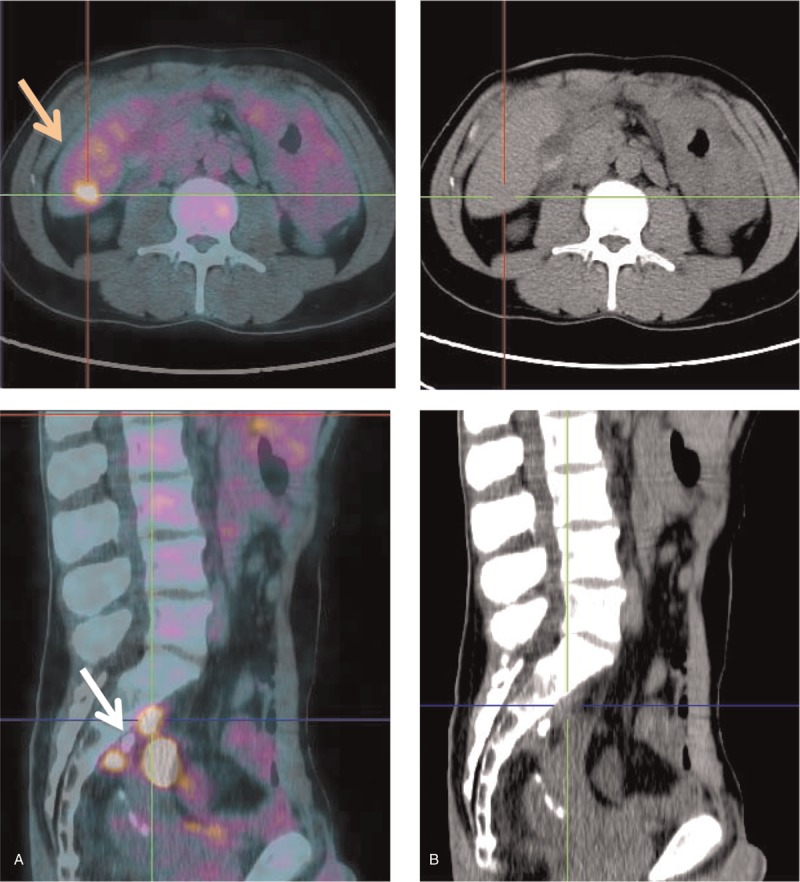
Axial (A) and sagittal (B) images: fused FDG-positron emission tomography/CT (left), CT alone (right). (A) New hepatic metastatic lesions (orange arrow). (B) Tumor implants (white arrow). CT = computed tomography, FDG = fluorodeoxyglucose.

In June 2015, he repeated PET–CT (Fig. 3), which revealed new hepatic metastatic lesions and abdominal-pelvic tumor implants, so he returned to previous scheme of CTX. After 6 cycles, with hematologic toxicity requiring 25% dose reduction of all drugs except bevacizumab and several delays, on January 2016 the CT scan showed progression disease.

Patient started second-line treatment with 5-FU, oxaliplatin, and leucovorin (25% reduction of 5-FU) with bevacizumab. CT scan after 6 cycles demonstrated new disease progression and started third-line CTX with FOLFIRI (75% dose of all drugs) plus cetuximab. On April of the current year, patient had new peritoneal disease progression and was admitted in our center with acute bowel occlusion and treated with conservative measures, but unfortunately he get worse and died 3 weeks later.

## Discussion

About half of the patients with CRC will have disease recurrence as peritoneal metastasis, and 10% of these will have isolated peritoneal disease.^2,3^

This case represents an example of metachronous peritoneal metastasis from primary rectal cancer and describes a multimodal and individual approach.

Regarding the treatment options undertaken, some considerations should be made. First, was this patient a candidate for neoadjuvant treatment at diagnosis instead of upfront surgery? If the tumor was a clinical T3 and/or positive for nodal disease, it should have; with neoadjuvant CRT the rates of local recurrence can be <6%.^4^ One thing is for sure: with the insufficient lymph nodes samples, at least adjuvant treatment should have been discussed.

Second, this patient with peritoneal disease at recurrence was selected for CS/HIPEC. But which patients should be candidates to undergo a CS/HIPEC approach? We don’t know; Goéré and colleagues published possible eligible criteria: ECOG (≤1), low peritoneal cancer index, absence of progression under CTX, no extra peritoneal metastasis (except up to 3 small liver metastasis potentially resectable), and no evidence of biliary or ureteral obstruction.^5^ Our patient was selected for its young age, excellent PS, no progression under CTX, and disease evaluated for complete resection, which also meet Goéré criteria.

Moreover, peritoneal carcinomatosis is found in advanced stage in most of the patients, due to absence of symptoms and because it is difficult to detect by imaging in early stage, which also make more challenging to achieve a complete resection.

Currently, there are phase II trials that support second-look surgery in patients with colon cancer at high risk for local recurrence or peritoneal metastases.^2,6^

Based on this rationale, Ripley et al designed a trial to evaluate if mandatory second-look surgery with CS/HIPEC would prolong OS compared with the standard of care (surveillance) in patients who have undergone curative surgery and show no evidence of disease, but who were at high risk for developing peritoneal carcinomatosis from CRC.^7^

These high-risk patients were described as having limited and synchronous peritoneal disease completely resected with the primary tumor, ovarian metastases, tumor perforation, T4 lesions, and emergency presentation with bleeding and obstruction.

The results of this study are still awaited, and at present, we do not have strong evidence-based data, so the authors do not recommend this proactive treatment strategy in rectal cancer.

Another promising approach in management of peritoneal carcinomatosis is delivering CTX into the peritoneal cavity as a pressurized normothermic aerosol via laparoscopy, known as PIPAC (pressurized intraperitoneal aerosol chemotherapy).^8^ This pressure application improves tumor drug uptake, and positive results with PIPAC with low-dose cisplatin and doxorubicin for patients with platin-resistant recurrent ovarian and gastric cancer have been published.^9,10^ In addition, a retrospective analysis presented the results of PIPAC with oxaliplatin in colorectal peritoneal metastasis.^11^ Objective tumor responses were observed in 71% of the patients (12 of the 17 patients), with minimal adverse effects, and mean survival after first PIPAC of 15.7 months. Further studies are necessary to evaluate the possible role of this strategy in CRC with peritoneal carcinomatosis.

We would like to emphasize the patient long survival, despite the poor prognosis and life expectancy <6 months estimated, in the presence of recurrence as peritoneal disease.^12^

Nowadays, the standard of care in the management of patients with metastatic CRC is the addition of biologic agents to CTX backbones as it is associated with improved OS that ranges to 25 to 28 months.^13^

Our patient received FOLFIRI/bevacizumab with significant imagiologic response on PET-fluorodeoxyglucose (FDG), so it was decided to restart the same CTX after second relapse. Several CT scans were negative for presence of disease so we chose PET-FDG for disease evaluation, mostly for 2 reasons: it provides a good overall accuracy in detecting peritoneal carcinomatosis, and helps in the selection of patients for surgery; however, our experience is important only when the implants are of great dimension.^14^

In conclusion, we highlight that patients treatment should be performed in experienced centers, in a multidisciplinary setting, in order to carefully select the mostly adequate approach for each patient.

## Acknowledgments

None.

## Conflicts of interest

The authors declare no conflicts of interest.

## Abbreviations

Abbreviations: 5-FU = 5-fluorouracil, CRC = colorectal cancer, CRT = chemoradiation therapy, CS/HIPEC = cytoreduction surgery with hyperthermic intraperitoneal chemotherapy, CT = computed tomography, CTX = chemotherapy, ECOG-PS = Eastern Cooperative Oncology Group performance status, FOLFIRI = 5-fluorouracil, leucovorin, irinotecan, MDT = multidisciplinary team, OS = overall survival, PET–CT = positron emission tomography–computed tomography, RT = radiotherapy.
